# Evaluation of herb-drug interaction of ambrisentan with shikonin based on UPLC-MS/MS

**DOI:** 10.1080/13880209.2021.1964544

**Published:** 2021-08-19

**Authors:** Tian Lan, Ping Fang, Xuemei Ye, Xia Lan, Ren-ai Xu

**Affiliations:** a The Quzhou Affiliated Hospital of Wenzhou Medical University, Quzhou People’s Hospital, Quzhou, Zhejiang, China; b The First Affiliated Hospital, Zhejiang University School of Medicine, Hangzhou, Zhejiang, China; c The First Affiliated Hospital of Wenzhou Medical University, Wenzhou, Zhejiang, China; d Chongqing University Cancer Hospital, Chongqing, China

**Keywords:** Inhibitory effect, (*S*)-4-hydroxymethyl ambrisentan, determination, microsomes, rat plasma

## Abstract

**Context:**

Ambrisentan is an oral endothelin-receptor antagonist (ERA). However, there is no report on the interaction between ambrisentan and shikonin.

**Objective:**

To investigate the effect of shikonin on ambrisentan metabolism *in vivo* and *in vitro*.

**Materials and methods:**

This study developed an ultra-performance liquid chromatography tandem mass spectrometry (UPLC-MS/MS) method for simultaneous determination of ambrisentan and (*S*)-4-hydroxymethyl ambrisentan in rat plasma. Twelve male Sprague-Dawley (SD) rats were divided into two groups (*n* = 6): the control group and shikonin (20 mg/kg) group. The pharmacokinetics of ambrisentan (2.5 mg/kg) were investigated after 30 min. Additionally, human and rat liver microsomes were used to investigate the herb-drug interaction.

**Results:**

The UPLC-MS/MS method was shown to be accurate, precise and reliable, and was successfully applied to the herb-drug interaction study of ambrisentan with shikonin. When co-administrated with 20 mg/kg shikonin, the *C*_max_ and AUC_(0–∞)_ of ambrisentan were significantly increased by 44.96 and 16.65%, respectively (*p* < 0.05). In addition, there were modest decreases in (*S*)-4-hydroxymethyl ambrisentan C_max_ and AUC_(0–∞)_ in the presence of shikonin (*p* < 0.05), which indicated that these results were in accordance with the inhibition of shikonin on ambrisentan metabolism. Moreover, enzyme kinetic study indicated that shikonin had an inhibitory effect on human and rat microsomes where the IC_50_ values of shikonin were 5.865 and 6.358 μM, respectively.

**Conclusions:**

Our study indicated that shikonin could inhibit ambrisentan metabolism. Further studies need to be carried out to verify whether similar interaction truly apply in humans and whether this interaction has clinical significance.

## Introduction

Pulmonary arterial hypertension (PAH) is a life-threatening, progressive disease characterized by ever-increasing pulmonary vascular resistance and sustained elevations in pulmonary artery pressure, which ultimately result in right ventricular failure and even premature death (Rich et al. 1987; Rubin 1997; Galie et al. 2008). Currently, endothelin-receptor antagonists (ERAs) are used commonly in the treatment of PAH (Rubin 1997; Humbert et al. 2004). Of them, ambrisentan is an orally active, nonsulfonamide, propanoic acid class of ERA, which can prevent the constriction or narrowing of blood vessels, thereby exerting beneficial effects in the treatment of PAH (Galie et al. 2005; Rubin et al. 2005). It is not only metabolized by hepatic phase glucuronidation, but also undergoes phase oxidative metabolism (Barst 2007; Vizza et al. 2012). The main metabolite (*S*)-4-hydroxymethyl ambrisentan is produced by cytochrome P450 (CYP) 3A4 (Croxtall and Keam 2008).

Shikonin, a kind of lipophilic hydroxynaphthoquinone, is mostly found in the root epidermis of at least 150 traditional medicinal species that belong to the Boraginaceae family (Assimopoulou et al. 2006; Hu et al. 2006; Albreht et al. 2009). It has anti-inflammatory, antithrombotic, antioxidant, antibacterial, antifungal, and antitumor properties (Tabata et al. 1975; Sankawa et al. 1977; Wang et al. 1994; Albreht et al. 2009). According to the pathophysiology, PAH can occur in association with diseases such as inflammatory, thrombus, tumour or cirrhosis of the liver (Humbert et al. 2004; Schermuly et al. 2011). Therefore, it is suggested that co-administration of ambrisentan with shikonin may occur when PAH patients also have other diseases that may be treated with shikonin. However, potential herb-drug interactions between ambrisentan and shikonin have not been reported in detail.

There are several studies about the determination of plasma ambrisentan concentrations by using liquid chromatography/tandem mass spectrometry (LC-MS/MS) (Nirogi et al. 2012; Lukram and Sharma 2014; Yokoyama et al. 2014; Garcia-Martinez et al. 2018; Tanaka et al. 2020; van de Velde et al. 2020). However, to date, there are only two published reports about the simultaneous determination of the concentrations of ambrisentan and its metabolite (*S*)-4-hydroxymethyl ambrisentan in plasma by LC-MS/MS (Enderle et al. 2015, 2017). However, limited availability of isotope-labeled internal standards, the complex sample preparation procedure (solid phase extraction) and long chromatographic run time (12 min) prohibited the use of both methods for pharmacokinetic and/or interaction studies. In this study, we develop a sensitive and validated ultra-performance liquid chromatography-tandem mass spectrometry (UPLC-MS/MS) method for the simultaneous determination of ambrisentan and (*S*)-4-hydroxymethyl ambrisentan in plasma. Meanwhile, this method was successfully applied to measure concentrations of ambrisentan and (*S*)-4-hydroxymethyl ambrisentan in an interaction study, which investigated the effect of shikonin on ambrisentan metabolism *in vivo* and *in vitro*.

## Materials and methods

### Materials

Ambrisentan and (*S*)-4-hydroxymethyl-ambrisentan were obtained from Toronto Research Chemicals (Toronto, Ontario, Canada). Midazolam used as internal standard (IS) was purchased from the Sigma-Aldrich (St. Louis, MO, USA). LC-grade acetonitrile, methanol and formic acid (FA, 98% purity) were purchased from Merck (Darmstadt, Germany). Ultrapure water was produced by the Millipore purification system (Millipore, Bedford, MA, USA). All of the other chemicals and solvents were of analytical grade.

### LC-MS/MS instrument and conditions

The liquid chromatographic separation was performed on a Waters ACQUITY UPLC BEH C18 column (2.1 × 50 mm, 1.7 µm) and column temperature was maintained at 40 °C. The initial mobile phase was consisted of 60% solvent A (water containing 0.1% formic acid) and 40% solvent B (acetonitrile) at the flow rate of 0.4 mL/min. The gradient elution was used as follows: 0–0.6 min linearly increased to 90% solvent B, 0.6–1.2 min maintained at 90% solvent B, and 1.2–1.8 min the solvent B was steeply reversed back to 40%. A subsequent re-equilibration time (2.2 min) should be performed before the next injection. The injection volume was 2 μL.

Samples were analyzed by ultra-performance liquid chromatography-tandem mass spectrometry (UPLC-MS/MS) using a Waters Acquity I-Class and a Waters XEVO TQS triple-quadrupole mass spectrometer (Waters Corp., Milford, MA, USA) with an electrospray ionization (ESI) source. A dynamic multiple reaction monitoring (MRM) method was developed to detect specific precursor and product ions of ambrisentan and its metabolite inside their retention time window (Table 1). Data acquisition was performed by Masslynx 4.1 software (Waters Corp.).

**Table 1.** MS parameters for ambrisentan, (*S*)-4-hydroxymethyl ambrisentan and midazolam.

Compound name	Precursor ion (*m/z*)	Product ion (*m/z*)	Collision energy (V)	Cone voltage (V)
Ambrisentan	379.24	347.28	5	10
(*S*)-4-Hydroxymethyl ambrisentan	395.11	363.17	5	30
Midazolam	326.20	291.0	30	60

### Preparation of calibration standards and quality control samples

Standard stock solutions of ambrisentan (1 mg/mL), (*S*)-4-hydroxymethyl-ambrisentan (1 mg/mL), and the IS (1 mg/mL) were separately prepared by dissolving in methanol. The IS working solution (500 ng/mL) was prepared daily by diluting its stock solution with methanol. Working solutions for calibration and controls were prepared by appropriate dilution of stock solutions with methanol. The calibration standards were prepared by adding 10 μL ambrisentan or (*S*)-4-hydroxymethyl ambrisentan working solutions with different concentrations into 90 μL drug-free rat plasma. The final concentrations of calibration standards were 100, 500, 1000, 2000, 5000, and 10,000 ng/mL for ambrisentan and 1, 5, 10, 50, 100, and 500 ng/mL for (*S*)-4-hydroxymethyl ambrisentan.

Quality control (QC) samples were prepared independently in the same way at three levels: 400, 4000, 8000 ng/mL for ambrisentan and 4, 40, 400 ng/mL for (*S*)-4-hydroxymethyl ambrisentan. All stock solutions, working solutions, calibration standards and QCs were preserved at −20 °C until analysis.

### Sample preparation

In a 1.5 mL polypropylene centrifuge tube, 10 μL midazolam (500 ng/mL) was added to 100 µL of plasma sample followed by the addition of 200 µL acetonitrile. After vortexing for 2.0 min, the tubes were centrifugated for 10 min at 13,000 rpm. To 100 µL supernatant, 100 µL water was added and mixed, then 2 µL was injected into the UPLC-MS/MS system for analysis.

### Method validation

Selectivity is a specialty of a method which can accurately quantify the target analyte by suppressing interference of endogenous compounds. It was evaluated by analyzing six different blank plasma samples from six rats, blank plasma spiked ambrisentan, (*S*)-4-hydroxymethyl ambrisentan and IS, as well as the rat plasma samples.

Six-point calibration curves (100–10,000 ng/mL for ambrisentan and 1–500 ng/mL for (*S*)-4-hydroxymethyl ambrisentan) in triplicate were measured and assayed on 3 days. The linearity for ambrisentan and (*S*)-4-hydroxymethyl ambrisentan were evaluated by the weighted (1/x^2^) least squares linear regression of the peak area ratios against concentrations. The lower limit of quantification (LLOQ) was defined as the lowest concentration on the calibration curves.

The matrix effect was evaluated by comparing the peak area ratio of analytes added into post-extracted plasma samples from six different drug-free rat plasma samples at three concentration levels (LQC, MQC, and HQC) with pure standard samples at corresponding concentrations. The extraction recovery was evaluated by the peak area ratios of extracted QC samples to those of post-extraction samples containing equivalent amount of the analytes.

The accuracy and precision of the method were assessed by analyzing three different concentrations of the QC samples in three separate days. Relative standard deviation (RSD, %) and the percentage of the measured concentration to the nominal concentration (relative error RE, %) were used to assess the accuracy and precision of the method. As required, variation should be within ± 15% for both precision and accuracy.

### Animals and *in vivo* pharmacokinetic analysis

Male Sprague-Dawley (SD) rats (250–280 g) were obtained from Laboratory Animal Centre of Wenzhou Medical University (Wenzhou, China). The rats were housed at a temperature of 25 °C and were acclimatized for 14 days in experimental conditions to minimize all influence.

For studying the effect of shikonin on the pharmacokinetic of ambrisentan, 12 SD rats were divided into two groups: the control group (group A, *n* = 6) and study group (group B, *n* = 6). Diet was prohibited for 12 h while water was not limited before administration. Shikonin was dissolved in oil and ambrisentan was suspended in 0.5% Carboxy Methyl Cellulose (CMC). Group A were treated with oil and Group B were treated with shikonin (20 mg/kg) by oral administration. Ambrisentan (2.5 mg/kg) was oral administrated half an hour later. Then, 0.3 mL blood samples were collected into heparinized tubes via the tail vein at time points of 0.083, 0.25, 0.5, 0.75, 1, 1.5, 2, 4, 8, 10, 12 and 24 h. Blood samples were centrifuged at 13,000 rpm for 10 min. The plasma samples were immediately separated from whole blood and stored at −80 °C until analysis.

### *In vitro* metabolism study

In order to study the effect of shikonin on the metabolism of ambrisentan in human and rat liver microsomes, shikonin (0.01, 0.1, 5, 10, 25, 50 and 100 μΜ) and ambrisentan (concentration at about the respective *K*_m_ value) were added to a 0.1 M Tris-HCl reaction mixture containing human liver microsomes or rat liver microsomes to determine the half-maximal inhibitory concentration (IC_50_).

The reactions were preincubated for 5 min at 37 °C, and then NADPH was added to start the reaction in a final volume of 200 μL. And incubations proceeded at 37 °C for 60 min. Reactions were terminated by cooling to −80 °C immediately. Midazolam (20 μL, with the concentration of 500 ng/mL) was added to the mixture followed by the addition of 0.4 mL of acetonitrile, which was then vortexed for 2.0 min and centrifuged at 13,000 rpm for 10 min. The supernatant was 1:1 diluted with water and 2 μL mixture was injected into the UPLC–MS/MS system for analysis. Incubations were performed in triplicate and the data are presented as the mean ± standard deviation.

## Results and discussion

### Method development

In order to obtain good peak symmetry, high detection sensitivity and shorten retention times, we had optimized many liquid chromatographic conditions, such as column type and mobile phase. Waters ACQUITY UPLC BEH C18 column (2.1 × 50 mm, 1.7 µm) with column temperature at 40 °C achieved effective separation and symmetrical peak shapes. In addition, various mobile phase combinations including acetonitrile, methanol and water with or without formic acid with altered flow rates were evaluated for optimum chromatographic retention of the analytes and IS. Finally, a mixture of acetonitrile and 0.1% formic acid in water with a gradient elution at flow rate of 0.4 mL/min was employed, which could obtain high detection sensitivity and shorten retention time. Moreover, mass parameters conditions were also optimized to obtain better resolution and higher response. At the conclusion of MS/MS optimization, the most intense fragment signal was used to quantify, and detailed information is presented in Table 1.

### Selectivity

**Figure 1.**
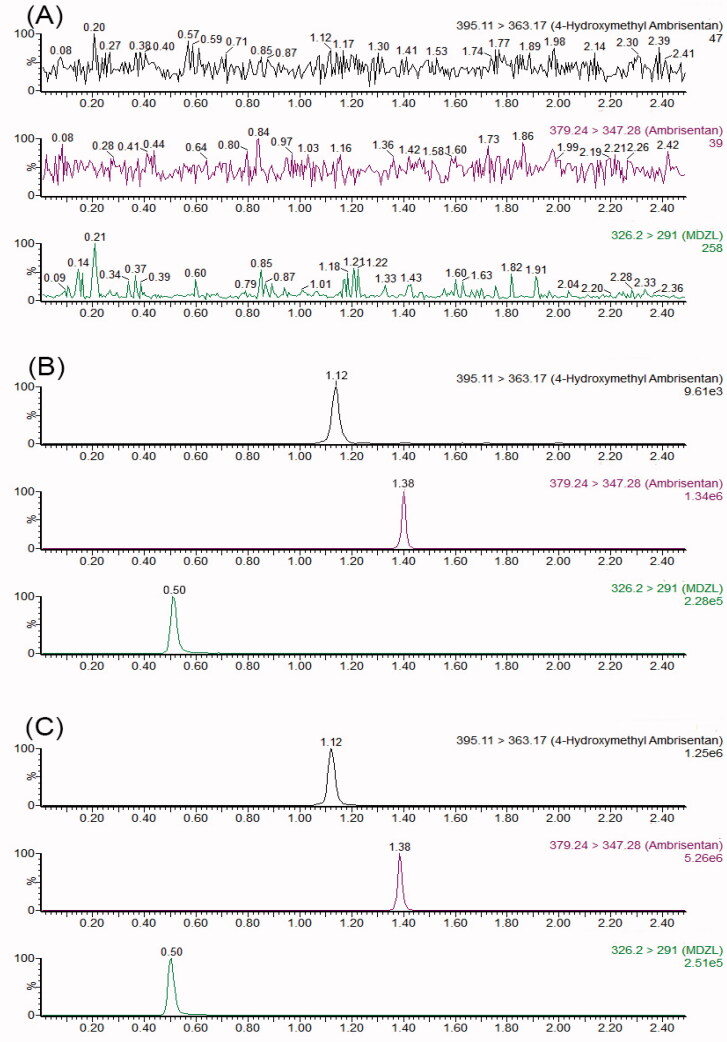
UPLC–MS/MS chromatograms of ambrisentan, its metabolite (*S*)-4-hydroxymethyl ambrisentan and IS. (A) A blank serum sample; (B) a blank plasma sample spiked with ambrisentan, (*S*)-4-hydroxymethyl ambrisentan and IS; (C) a rat plasma sample.

The selectivity of the method was examined by analyzing samples obtained from blank plasma (Figure 1(A)), blank plasma spiked with analytes (Figure 1(B)) and a rat plasma sample (Figure 1(C)). Compared with the blank plasma, there was no interference from endogenous substances.

### Linearity and LLOQ

Linear regression analysis was used to construct the calibration curves over the concentration range of 100–10,000 ng/mL for ambrisentan and 1–500 ng/mL for (*S*)-4-hydroxymethyl ambrisentan, respectively. The regression coefficients *R*^2^ of two calibration curves were both >0.99, which indicated that they had good linearity. The LLOQ of ambrisentan and (*S*)-4-hydroxymethyl ambrisentan were 100 ng/mL and 1 ng/mL with the RSD within ±20% and RE did not exceed ±20% of the nominal concentration.

### Matrix effect and extraction recovery

The matrix effect for ambrisentan at concentrations of 400, 4000 and 8000 ng/mL were measured to be 102.34, 95.82, and 116.47% (*n* = 6), respectively (Table 2). The matrix effect for (*S*)-4-hydroxymethyl ambrisentan at concentrations of 4, 40, and 400 ng/mL were measured to be 118.49, 108.56, and 109.61% (*n* = 6), respectively. The matrix effect for the IS (500 ng/mL) was 88.66% (*n* = 6). The results showed the matrix effect values were within the acceptable limits (85–115%), which indicated that the matrix effect is negligent in our study.

**Table 2.** Precision, accuracy, matrix effect (ME) and recovery for analytes of QC sample in rat plasma (*n* = 6).

	Concentration	Intra-day			Inter-day				
Analytes	ng/mL	Mean ± SD	RSD (%)	RE (%)	Mean ± SD	RSD (%)	RE (%)	Recovery (%)	ME (%)
	4	4.37 ± 0.19	4.25	9.33	4.33 ± 0.11	2.52	8.22	104.16	118.49
4-(*S*)-Hydroxymethyl ambrisentan	40	41.35 ± 1.44	3.49	3.37	41.63 ± 0.49	1.19	4.08	105.89	108.56
	400	420.91 ± 15.12	3.59	5.23	426.67 ± 6.18	1.45	6.67	109.62	109.61
	400	420.65 ± 33.90	8.06	5.16	421.77 ± 18.59	4.41	5.44	93.66	102.34
Ambrisentan	4000	3968.10 ± 277.363	6.99	−0.80	4107.74 ± 128.31	3.12	2.69	91.51	95.82
	8000	8209.13 ± 151.21	1.84	2.61	7822.28 ± 337.01	4.31	−2.22	105.57	116.47

The recovery of ambrisentan and (*S*)-4-hydroxymethyl ambrisentan were between 91.51 and 109.62% over three QC sample levels. It proved that the method to measure ambrisentan and (*S*)-4-hydroxymethyl ambrisentan was reliable, accurate and reproducible.

### Accuracy and precision

The accuracy and precision values were determined via six replicates of QC samples at three concentrations. The results are summarized in Table 2. RSD was used to express intra-day and inter-day precision, and RE was used to express intra-day and inter-day accuracy. The method was repeatable and reproducible since RSD was below 8.06% and RE was ranged from −2.22 to 9.33% for all the investigated concentrations of analytes in rat plasma.

### Effects of shikonin on the metabolism of ambrisentan *in vivo* and *in vitro*

**Figure 2.**
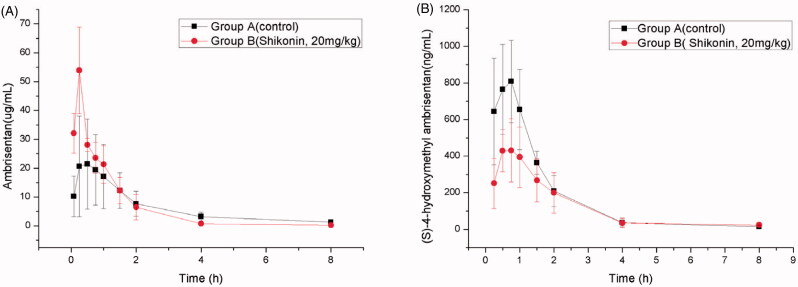
Mean ± SD concentration–time curve of ambrisentan (A) and (*S*)-4-hydroxymethyl ambrisentan (B) in two groups (*n* = 6 each group).

The mean pharmacokinetic parameters of ambrisentan administered alone (group A) and in combination with shikonin (group B, 20 mg/kg shikonin) were calculated by DAS 3.0, and the statistical analysis results are presented in Tables 3 and 4, respectively. Mean plasma concentration–time curves of ambrisentan and (*S*)-4-hydroxymethyl ambrisentan in the two groups are presented in Figure 2.

**Table 3.** The main pharmacokinetic parameters of ambrisentan in rat plasma in two groups (*n* = 6).

Parameters	Group A	Group B
AUC_(0–t)_ (μg/L·h)	41,308.92 ± 19,275.09	53,368.89 ± 7,683.32*
AUC_(0–∞)_ (μg/L·h)	44,893.16 ± 22,304.94	53,861.05 ± 7,773.54*
*t*_1/2_z (h)	0.92 ± 0.42	0.55 ± 0.11
*T*_max_ (h)	0.60 ± 0.55	0.22 ± 0.08
V_z_/F (L/kg)	0.09 ± 0.06	0.04 ± 0.01
CL_z_/F (L/h/kg)	0.07 ± 0.03	0.05 ± 0.01
*C*_max_ (μg/L)	31,474.53 ± 18,255.05	57,182.51 ± 14,900.22*

Group A: control group; Group B: a single dose of 20 mg/kg shikonin. *p < 0.05.

**Table 4.** The main pharmacokinetic parameters of (*S*)-4-hydroxymethyl ambrisentan in rat plasma in two groups (*n* = 6).

Parameters	Group A	Group B
AUC_(0–t)_ (μg/L·h)	1,383.75 ± 329.16	961.42 ± 372.20*
AUC_(0–∞)_ (μg/L·h)	1,417.66 ± 343.58	994.23 ± 384.29*
*t*_1/2_z (h)	1.59 ± 0.26	1.25 ± 0.65
*T*_max_ (h)	0.55 ± 0.21	0.65 ± 0.22
V_z_/F (L/kg)	4.20 ± 1.05	4.91 ± 2.51
CL_z_/F (L/h/kg)	1.85 ± 0.46	2.86 ± 1.17
*C*_max_ (μg/L)	883.37 ± 221.66	482.91 ± 141.83*

Group A: control group; Group B: a single dose of 20 mg/kg shikonin. *p < 0.05.

The mean plasma concentration–time curve showed that it had the higher concentration of ambrisentan in Group B, which was given shikonin 20 mg/kg (Figure 2(A)), while it had the lower concentration of (*S*)-4-hydroxymethyl ambrisentan in Group B (Figure 2(B)), which indicated that shikonin could decrease the metabolism rate of ambrisentan *in vivo* after the single-dose treatment. When co-administered with shikonin, the *C*_max_ and AUC_(0–∞)_ of ambrisentan were significantly increased by 44.96 and 16.65%, respectively (*p* < 0.05). In addition, the *T*_max_ and *t*_1/2_ of ambrisentan were slightly changed compared to those of the control group. And, there were modest decreases in (*S*)-4-hydroxymethyl ambrisentan *C*_max_ and AUC_(0–∞)_ in the presence of shikonin (*p* < 0.05), which indicated that these results were in accordance with the inhibition of shikonin on ambrisentan metabolism.

**Figure 3.**
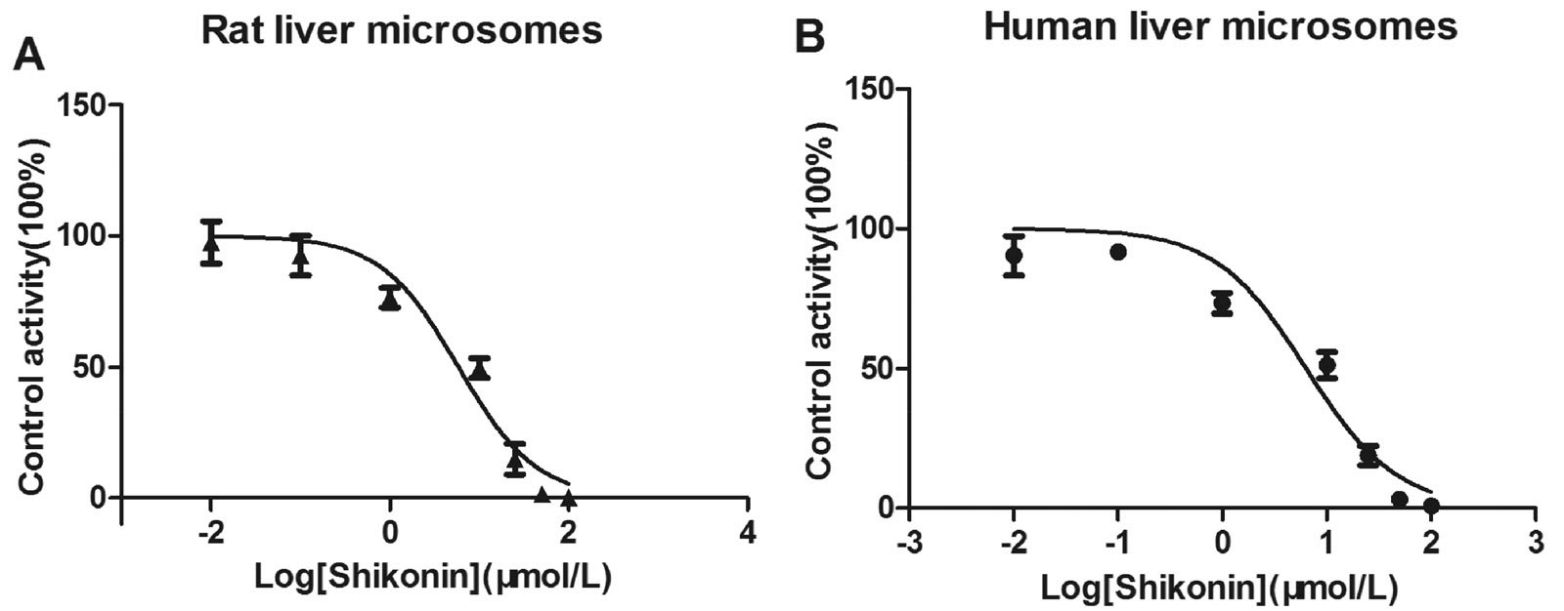
Various concentrations of shikonin for half-maximal inhibitory concentration (IC_50_) in the activity of (A) rat liver microsomes and (B) human liver microsomes. Values are mean ± SD, *n* = 3.

Moreover, as shown in Figure 3, the IC_50_ for inhibition activity in human liver microsomes was 5.865 μM, while it was 6.358 μM in rat liver microsomes. The results indicated that shikonin could inhibit the metabolism of ambrisentan both in human and rat liver microsomes.

According to previous reports, ambrisentan had little effect on hepatic CYP450 induction or inhibition. For example, rifampicin, a potent inducer and substrate of CYP3A4, had no clinically effect on pharmacokinetics of ambrisentan (Harrison et al. 2010). And ketoconazole, which is known as a strong inhibitor of CYP3A4, also had no clinically significant effect on the pharmacokinetics of ambrisentan (Richards et al. 2009). Furthermore, it has been reported that the CYP3A4 and OATP1B1 inhibitor clarithromycin increased ambrisentan exposure but clinically likely to be irrelevant (Markert et al. 2013). However, in our study, the increase of ambrisentan exposure in the presence of shikonin indicated that the CYP3A4 inhibition by shikonin could be a potential cause (Tang et al. 2017). Most frequent adverse events of patients receiving ambrisentan were reported peripheral edoema, headache, dizziness and nasal congestion (Elshaboury and Anderson 2013). These side effects were considered mild to moderate in nature. Thus, in the clinic, the combination of ambrisentan and shikonin should be avoided or monitored, because the pharmacokinetics of ambrisentan have been altered significantly. Therefore, exposure to ambrisentan would be significantly increased and its associated side effects would be more serious and frequent. Finally, further studies about the effect of shikonin on ambrisentan metabolism in clinical studies and its inhibitory mechanism should be further investigated.

## Conclusions

A rapid and sensitive method for the quantification of ambrisentan and (*S*)-4-hydroxymethyl ambrisentan in rat plasma by UPLC-MS/MS was developed and validated according to commonly accepted criteria. Moreover, the UPLC-MS/MS method was also applied to the herb-drug interaction study of ambrisentan with shikonin *in vivo* and *in vitro*. The results revealed that shikonin has a potential inhibitory effect on the metabolism of ambrisentan.

## References

[CIT0001] Albreht A, Vovk I, Simonovska B, Srbinoska M. 2009. Identification of shikonin and its ester derivatives from the roots of *Echium italicum* L. J Chromatogr A. 1216(15):3156–3162. 19233376 10.1016/j.chroma.2009.01.098

[CIT0002] Assimopoulou AN, Karapanagiotis I, Vasiliou A, Kokkini S, Papageorgiou VP. 2006. Analysis of alkannin derivatives from *Alkanna* species by high-performance liquid chromatography/photodiode array/mass spectrometry. Biomed Chromatogr. 20(12):1359–1374. 17080496 10.1002/bmc.705

[CIT0003] Barst RJ. 2007. A review of pulmonary arterial hypertension: role of ambrisentan. Vasc Health Risk Manag. 3(1):11–22. 17583171 PMC1994051

[CIT0004] Croxtall JD, Keam SJ. 2008. Ambrisentan. Drugs. 68(15):2195–2204. 18840007 10.2165/00003495-200868150-00008

[CIT0005] Elshaboury SM, Anderson JR. 2013. Ambrisentan for the treatment of pulmonary arterial hypertension: improving outcomes. Patient Prefer Adherence. 7:401–409. 23674888 10.2147/PPA.S30949 PMC3652514

[CIT0006] Enderle Y, Meid AD, Friedrich J, Grunig E, Wilkens H, Haefeli WE, Burhenne J. 2015. Dried blood spot technique for the monitoring of ambrisentan, bosentan, sildenafil, and tadalafil in patients with pulmonary arterial hypertension. Anal Chem. 87(24):12112–12120. 26583764 10.1021/acs.analchem.5b03077

[CIT0007] Enderle Y, Witt L, Wilkens H, Grunig E, Haefeli WE, Burhenne J. 2017. Simultaneous quantification of endothelin receptor antagonists and phosphodiesterase 5 inhibitors currently used in pulmonary arterial hypertension. J Pharm Biomed Anal. 143:291–298. 28628863 10.1016/j.jpba.2017.05.052

[CIT0008] Galie N, Badesch D, Oudiz R, Simonneau G, McGoon MD, Keogh AM, Frost AE, Zwicke D, Naeije R, Shapiro S, et al. 2005. Ambrisentan therapy for pulmonary arterial hypertension. J Am Coll Cardiol. 46(3):529–535. 16053970 10.1016/j.jacc.2005.04.050

[CIT0009] Galie N, Rubin L, Hoeper M, Jansa P, Al-Hiti H, Meyer G, Chiossi E, Kusic-Pajic A, Simonneau G. 2008. Treatment of patients with mildly symptomatic pulmonary arterial hypertension with bosentan (EARLY study): a double-blind, randomised controlled trial. Lancet. 371(9630):2093–2100. 18572079 10.1016/S0140-6736(08)60919-8

[CIT0010] Garcia-Martinez S, Rico E, Casal E, Grisalena A, Alcaraz E, King N, Leal N, Navarro I, Campanero MA. 2018. Bionalytical validation study for the determination of unbound ambrisentan in human plasma using rapid equilibrium dialysis followed by ultra performance liquid chromatography coupled to mass spectrometry. J Pharm Biomed Anal. 150:427–435. 29289894 10.1016/j.jpba.2017.12.030

[CIT0011] Harrison B, Magee MH, Mandagere A, Walker G, Dufton C, Henderson LS, Boinpally R. 2010. Effects of rifampicin (rifampin) on the pharmacokinetics and safety of ambrisentan in healthy subjects: a single-sequence, open-label study. Clin Drug Investig. 30(12):875–885. 10.2165/11539110-000000000-00000 20923245

[CIT0012] Hu Y, Jiang Z, Leung KS, Zhao Z. 2006. Simultaneous determination of naphthoquinone derivatives in Boraginaceous herbs by high-performance liquid chromatography. Anal Chim Acta. 577(1):26–31. 17723649 10.1016/j.aca.2006.06.031

[CIT0013] Humbert M, Sitbon O, Simonneau G. 2004. Treatment of pulmonary arterial hypertension. N Engl J Med. 351(14):1425–1436. 15459304 10.1056/NEJMra040291

[CIT0014] Lukram OK, Sharma R. 2014. High-performance liquid chromatography tandem mass spectrometry method for quantification of endothelin receptor antagonist drug, ambrisentan, in human plasma and its application in a pharmacokinetic study. Biomed Chromatogr. 28(8):1147–1155. 24616031 10.1002/bmc.3136

[CIT0015] Markert C, Hellwig R, Burhenne J, Hoffmann MM, Weiss J, Mikus G, Haefeli WE. 2013. Interaction of ambrisentan with clarithromycin and its modulation by polymorphic SLCO1B1. Eur J Clin Pharmacol. 69(10):1785–1793. 23748747 10.1007/s00228-013-1529-1

[CIT0016] Nirogi R, Kandikere V, Komarneni P, Aleti R, Padala N, Kalaikadhiban I. 2012. LC-ESI-MS/MS method for quantification of ambrisentan in plasma and application to rat pharmacokinetic study. Biomed Chromatogr. 26(10):1150–1156. 22222607 10.1002/bmc.2670

[CIT0017] Rich S, Dantzker DR, Ayres SM, Bergofsky EH, Brundage BH, Detre KM, Fishman AP, Goldring RM, Groves BM, Koerner SK. 1987. Primary pulmonary hypertension. A national prospective study. Ann Intern Med. 107(2):216–223. 3605900 10.7326/0003-4819-107-2-216

[CIT0018] Richards DB, Walker GA, Mandagere A, Magee MH, Henderson LS. 2009. Effect of ketoconazole on the pharmacokinetic profile of ambrisentan. J Clin Pharmacol. 49(6):719–724. 19389876 10.1177/0091270009335870

[CIT0019] Rubin LJ. 1997. Primary pulmonary hypertension. N Engl J Med. 336(2):111–117. 8988890 10.1056/NEJM199701093360207

[CIT0020] Rubin LJ, Dufton C, Gerber MJ. 2005. Ambrisentan for pulmonary arterial hypertension. Future Cardiol. 1(4):425–432. 19804142 10.2217/14796678.1.4.425

[CIT0021] Sankawa U, Ebizuka Y, Miyazaki T, Isomura Y, Otsuka H. 1977. Antitumor activity of shikonin and its derivatives. Chem Pharm Bull. 25(9):2392–2395. 10.1248/cpb.25.2392 589729

[CIT0022] Schermuly RT, Ghofrani HA, Wilkins MR, Grimminger F. 2011. Mechanisms of disease: pulmonary arterial hypertension. Nat Rev Cardiol. 8(8):443–455. 21691314 10.1038/nrcardio.2011.87 PMC7097518

[CIT0023] Tabata M, Mizukami H, Naoe S, Konoshima M. 1975. Antimicrobial activity of *Lithospermum erythrorhizon* callus cultures. Yakugaku Zasshi. 95(11):1376–1379. 1240939 10.1248/yakushi1947.95.11_1376

[CIT0024] Tanaka S, Uchida S, Hakamata A, Miyakawa S, Odagiri K, Inui N, Watanabe H, Namiki N. 2020. Simultaneous LC-MS analysis of plasma concentrations of sildenafil, tadalafil, bosentan, ambrisentan, and macitentan in patients with pulmonary arterial hypertension. Pharmazie. 75(6):236–239. 32539916 10.1691/ph.2020.0021

[CIT0025] Tang S, Chen A, Zhou X, Zeng L, Liu M, Wang X. 2017. Assessment of the inhibition risk of shikonin on cytochrome P450 via cocktail inhibition assay. Toxicol Lett. 281:74–83. 28941798 10.1016/j.toxlet.2017.09.014

[CIT0026] van de Velde D, Bahmany S, Hitzerd E, van Domburg B, Versmissen J, Danser AHJ, Koch BCP. 2020. Simultaneous quantification of ambrisentan, macitentan and sitaxentan in human plasma using UPLC-MS/MS. Biomed Chromatogr. 34(3):e4787. 31875652 10.1002/bmc.4787

[CIT0027] Vizza CD, Fedele F, Pezzuto B, Rubin LJ. 2012. Safety and efficacy evaluation of ambrisentan in pulmonary y hypertension. Expert Opin Drug Saf. 11(6):1003–1011. 22861496 10.1517/14740338.2012.714770

[CIT0028] Wang WJ, Bai JY, Liu DP, Xue LM, Zhu XY. 1994. The antiinflammatory activity of shikonin and its inhibitory effect on leukotriene B4 biosynthesis. Yao Xue Xue Bao. 29(3):161–165. 8079645

[CIT0029] Yokoyama Y, Tomatsuri M, Hayashi H, Hirai K, Ono Y, Yamada Y, Todoroki K, Toyo'oka T, Yamada H, Itoh K. 2014. Simultaneous microdetermination of bosentan, ambrisentan, sildenafil, and tadalafil in plasma using liquid chromatography/tandem mass spectrometry for pediatric patients with pulmonary arterial hypertension. J Pharm Biomed Anal. 89:227–232. 24309556 10.1016/j.jpba.2013.11.007

